# Splenial white matter integrity is associated with memory impairments in posterior cortical atrophy

**DOI:** 10.1093/braincomms/fcab060

**Published:** 2021-04-28

**Authors:** Margot Juliëtte Overman, Giovanna Zamboni, Christopher Butler, Samrah Ahmed

**Affiliations:** 1 Research Institute for the Care of Older People (RICE), Bath BA1 3NG, UK; 2 MRC Cognition and Brain Sciences Unit, University of Cambridge, Cambridge CB2 7EF, UK; 3 Dipartimento di Scienze Biomediche, Metaboliche e Neuroscienze, Università di Modena e Reggio Emilia, Modena, Italy; 4 Center for Neuroscience and Neurotechnology, Università di Modena e Reggio Emilia, Modena, Italy; 5 Nuffield Department of Clinical Neuroscience, University of Oxford, Oxfordshire OX3 9DU, UK; 6 Department of Brain Sciences, Imperial College London, London SW7 2AZ, UK; 7 Departamento de Neurología, Pontificia Universidad Católica de Chile, Santiago, Chile; 8 School of Psychology and Clinical Language Sciences, University of Reading, Reading RG6 6ES, UK

**Keywords:** posterior cortical atrophy, diffusion tensor imaging, white matter, memory, splenium

## Abstract

Posterior cortical atrophy is an atypical form of Alzheimer’s disease characterized by visuospatial impairments and predominant tissue loss in the posterior parieto-occipital and temporo-occipital cortex. Whilst episodic memory is traditionally thought to be relatively preserved in posterior cortical atrophy, recent work indicates that memory impairments form a common clinical symptom in the early stages of the disease. Neuroimaging studies suggest that memory dysfunction in posterior cortical atrophy may originate from atrophy and functional hypoconnectivity of parietal cortex. The *structural* connectivity patterns underpinning these memory impairments, however, have not been investigated. This line of inquiry is of particular interest, as changes in white matter tracts of posterior cortical atrophy patients have been shown to be more extensive than expected based on posterior atrophy of grey matter. In this cross-sectional diffusion tensor imaging MRI study, we examine the relationship between white matter microstructure and verbal episodic memory in posterior cortical atrophy. We assessed episodic memory performance in a group of posterior cortical atrophy patients (*n* = 14) and a group of matched healthy control participants (*n* = 19) using the Free and Cued Selective Reminding Test with Immediate Recall. Diffusion tensor imaging measures were obtained for 13 of the posterior cortical atrophy patients and a second control group of 18 healthy adults. Patients and healthy controls demonstrated similar memory encoding performance, indicating that learning of verbal information was preserved in posterior cortical atrophy. However, retrieval of verbal items was significantly impaired in the patient group compared with control participants. As expected, tract-based spatial statistics analyses showed widespread reductions of white matter integrity in posterior cortical regions of patients compared with healthy adults. Correlation analyses indicated that poor verbal retrieval in the patient group was specifically associated with microstructural damage of the splenium of the corpus callosum. Post-hoc tractography analyses in healthy controls demonstrated that this splenial region was connected to thalamic radiations and the retrolenticular part of the internal capsule. These results provide insight into the brain circuits that underlie memory impairments in posterior cortical atrophy. From a cognitive perspective, we propose that the association between splenial integrity and memory dysfunction could arise indirectly via disruption of attentional processes. We discuss implications for the clinical phenotype and development of therapeutic aids for cognitive impairment in posterior cortical atrophy.

## Teaser

Using diffusion tensor imaging data, Overman *et al*. identified a specific relationship between white matter integrity of the splenium and memory retrieval in posterior cortical atrophy. These results fit within a framework in which memory deficits in posterior cortical atrophy are underpinned by disruption of attentional pathways.

## Introduction

Posterior cortical atrophy (PCA) is a neurodegenerative syndrome characterized by prominent impairments of visuospatial abilities, independent from ocular disease. PCA is most commonly associated with Alzheimer’s disease neuropathology, although some patients present with features more closely resembling Lewy body dementia, corticobasal degeneration or prion disease.^1^^,^^2^ Tissue loss and hypometabolism resulting from these processes are predominantly observed in posterior parieto-occipital and temporo-occipital cortex.^3–6^ In contrast to the amnestic presentation of Alzheimer’s disease, episodic memory is traditionally thought to be relatively preserved in PCA until later stages of the disease.^7^^,^^8^ However, recent work has shown that memory processes are compromised in the majority of PCA patients, and form an oft-reported early clinical symptom.^9–11^ The emerging profile of memory impairment is one of decreased encoding and retrieval of verbal information.^10^^,^^12^ Recognition and sensitivity to cues in memory retrieval appear to be less affected, with PCA patients achieving scores within the normal range when cues to recall are utilised.^13^^,^^14^

Episodic memory dysfunction is classically associated with the medial temporal lobe circuitry and has long been considered the hallmark feature of typical Alzheimer’s disease,^15^^,^^16^ which is characterized by impaired storage. In contrast, neuroimaging research consistently shows that the medial temporal lobe system is comparatively intact in the early to moderate stages of PCA.^6^^,^^8^^,^^17^^,^^18^ Instead, research suggests that the neural underpinnings of memory impairment in PCA originate in the posterior parietal neural networks of the brain. A recent voxel-based morphometry study demonstrated that impairments of immediate recall, delayed recall and recognition on a verbal memory task were associated with reduced grey matter density in the left postcentral gyrus in PCA patients.^12^ Rather than being implicated in memory performance, this region has previously been identified as a node of the frontoparietal cortical network with a role in orienting attention.^19^ Medial temporal regions were largely spared and did not correlate with memory performance. Resting-state analyses of PCA patients further indicated that reduced verbal recall performance was related to hypoconnectivity between the insula and the dorsal attention network, which encompasses the intraparietal sulcus and superior parietal lobule.^14^ Although the default mode network, which is typically disrupted in Alzheimer’s disease, also showed hypoconnectivity in PCA patients, this alteration was not associated with impaired performance on the memory task.

Longstanding views of the parietal lobe have traditionally ascribed its roles in support of planning and control of movement, attention to spatial information and construction.^20–22^ More recent neuroimaging studies, however, suggest that the posterior parietal cortex may also contribute to the neurocircuitry of episodic memory and, in particular, to encoding and retrieval mechanisms (for reviews, see Refs.^23–25^). Specifically, differential parietal activations have been observed for recognition of previously encountered items versus new items,^26–30^ recollection- and familiarity-driven recognition,^31–33^ and orienting retrieval to specific aspects of memory (e.g. source versus item^34^^,^^35^) Lesion studies provide convergent evidence for a salient role of parietal cortex in memory functioning. Significant impairments in spontaneous but not cued recall of autobiographical memories were reported in two patients with bilateral lesions in the inferior and superior parietal lobules as well as the intraparietal sulcus.^36^ Similarly, five patients with focal lesions in parietal cortex demonstrated significant impairments on verbal recall and recognition tasks on neuropsychological testing.^37^ In a large-scale study of 48 stroke patients with posterior parietal damage, significant associations were found between angular gyrus damage and cued recall on a verbal paired associates learning task.^38^ Damage to parietal cortex can result in specific memory retrieval deficits which differ from the dense amnesia observed after hippocampal damage.^39^

The underlying mechanisms linking these posterior cortical regions to memory remain under debate (for reviews, see Refs.^23^^,^^40^). One candidate hypothesis, entertained by a number of researchers previously,^23^^,^^41^ would propose that the lateral parietal cortex supports attentional processes during memory encoding and retrieval. The Attention to Memory (AtoM) model^24^^,^^42^ builds on prior theories of attention, which suggest that a dorsal frontoparietal system guides bottom-up attention whereas a ventral frontoparietal network is responsible for directing top-down visual attention.^22^ The AtoM postulates that these attentional mechanisms can be extended to the memory domain. Thus, a dorsal attention network with key nodes in the superior parietal lobe and intraparietal sulcus is presumed to mediate goal-directed attention to memories (i.e. top-down attention), whereas a ventral network encompassing the inferior parietal lobe including the temporoparietal junction and angular gyrus controls reflexive capture of attention to salient memory cues (i.e. bottom-up attention). Similar to this hypothesis, Gilmore et al.^43^ posit that a reliable parietal memory network which largely depends on bottom-up attentional capture of stimuli can be identified from the fMRI literature, consisting of the precuneus, mid-cingulate gyrus and the posterior inferior lobule/dorsal angular gyrus. Disrupted attentional processes could therefore be a driving force behind the memory deficits observed in PCA, with atrophy and functional hypoconnectivity of parietal cortex in PCA.^12^^,^^14^

In the present study, we aimed to further explore the neurocognitive underpinnings of memory impairment in PCA. Currently, no study has investigated *structural* connectivity changes in posterior neural networks which may contribute to memory functioning in PCA, yet there are compelling reasons to add this line of inquiry. Diffusion tensor imaging (DTI) studies have indicated that PCA is associated with widespread changes in white matter tracts, which are more extensive than expected when based on the posterior atrophy observed in structural imaging.^44^ It is therefore possible that reduced integrity of these tracts, and consequent disconnection of cognitive networks, may contribute to the memory symptoms observed in PCA, distinct from the effects of grey matter atrophy. Cross-sectional and longitudinal studies in PCA have identified white matter damage in the corpus callosum, cingulum, fornix, parietal lobe, and occipital lobe, as well as major tracts including the superior longitudinal fasciculus (SLF), inferior longitudinal fasciculus (ILF), inferior fronto-occipital fasciculus and parahippocampal tracts (see Table 1 for summary). Compared to typical Alzheimer’s disease, PCA showed greater involvement of the SLF, posterior cingulate, splenium of the corpus callosum, and posterior ILF and inferior fronto-occipital fasciculus.^45^ An examination of the memory neuroimaging literature reveals that many of the regions which are compromised in PCA have also been associated with memory performance in healthy adults (see Table 2). At present, however, it is unknown whether microstructural changes in these tracts contribute to the memory impairments observed in PCA.

**Table 1:** Tracts and white matter regions demonstrating degeneration in patients with Posterior Cortical Atrophy (PCA)

First author	Journal	PCA patient sample size	Analysis method	Outcome measure	Tracts/regions identified
Agosta^44^	*NeuroImage: Clinical*	21	Probabilistic tractography	FA, MD, AxD, and RD	Cingulum Corpus callosum, body Corpus callosum, splenium ILF, left SLF, bilateral
Caso^46^	*Radiology*	13	ROI-based TBSS	FA	Cingulum, posterior Corpus callosum IFOF ILF Parahippocampal tracts SLF
Cerami^3^	*Journal of Alzheimer's Disease*	6	Whole-brain TBSS	FA, MD, AxD, and RD	Cingulum bundle Corpus callosum, genu Corpus callosum, splenium Forceps minor Fornix IFOF ILF SLF Thalamic radiations Uncinate fasciculus
Duning^47^	*Journal of Neurology, Neurosurgery, and Psychiatry*	1	ROI-based TBSS	FA	Occipital lobe Parietal lobes
Glick-Shames^48^	*Brain Imaging and Behavior*	10	Probabilistic tractography	FA, AxD, and RD	Optic radiations Splenial fibers
Madhavan^45^	*Journal of Alzheimer's* *Disease*	18	Whole-brain voxel-based analysis	FA, MD, AxD, and RD	Cingulum Corpus callosum, body Corpus callosum, splenium Fornix IFOF ILF Internal capsule SLF Thalamic radiation, left posterior Uncinate fasciculus
Migliaccio^49^	*Cortex*	1	Tractography	FA, MD, AxD, and RD	Corpus callosum IFOF ILF SLF, fronto-parietal
Migliaccio^50^	*Alzheimer's & Dementia*	13	Voxel-based morphometry analysis	WM atrophy	Cingulum, posterior Corpus callosum, posterior Occipital region, bilateral Parietal region, bilateral Temporal region, bilateral
Migliaccio^51^	*Neurobiology of Aging*	7	Tractography and voxel-based morphometry analysis	FA, MD, AxD, and RD WM atrophy	*Tractography* Corpus callosum IFOF, bilateral ILF, left SLF, right fronto-parietal *Voxel-based morphometry* Bilateral ventral occipitotemporal region
Millington^52^	*NeuroImage: Clinical*	10	Whole-brain and ROI-based TBSS	FA and MD	Corpus callosum, genu Corpus callosum, splenium Occipital lobe
Yoshida^53^	*European Neurology*	1	ROI-based TBSS	FA	Splenium of the corpus callosum

ADC, apparent diffusivity coefficient; AxD, axial diffusivity; FA, fractional anisotropy; IFOF, inferior fronto-occipital fasciculus; ILF, inferior longitudinal fasciculus; MD, mean diffusivity; RD, radial diffusivity; ROI, region-of-interest; SLF, superior longitudinal fasciculus; TBSS, tract-based spatial statistics.

**Table 2:** Overview of neuroimaging studies of healthy adults demonstrating relationships between memory performance and white matter structures consistently affected by PCA

Structure	First author	Memory type	Imaging outcome measure	Direction of correlation
Cingulum	Alm^54^	Verbal episodic memory	MD and RD	↑
	Ezzati^55^	Verbal episodic memory	FA	↑
	Sasson^56^	Verbal and visual episodic memory	ADC	↓
	Sasson^57^	Verbal and visual episodic memory	ADC and AxD	↓
ILF	Hodgetts^58^	Semantic aspects of autobiographical memory	MD	↓
	Sasson^57^	Verbal and visual episodic memory	FA and RD	↑
SLF	Begre^59^	Visual episodic memory	Intervoxel coherence	↑
	Sasson^57^	Verbal and visual episodic memory	ADC and RD	↑
Splenium of the corpus callosum	Begre^59^	Visual episodic memory	Intervoxel coherence	↑
	Cox^60^	Verbal episodic memory	FA	↑
	Voineskos^61^	Verbal and visual episodic memory	FA	↑
Uncinate fasciculus	Alm^54^	Verbal episodic memory	White matter volume	↑
	Metzler-Baddeley^62^	Verbal episodic memory	FA	↑
	Niogi^63^	Verbal episodic memory	FA	↑
	Sasson^57^	Verbal and visual episodic memory	ADC	↓

↑ indicates a positive correlation, ↓ indicates a negative correlation.

ADC, apparent diffusivity coefficient; AxD, axial diffusivity; FA, fractional anisotropy; ILF, inferior longitudinal fasciculus; MD, mean diffusivity; RD, radial diffusivity; SLF, superior longitudinal fasciculus.

The aim of this study, therefore, is to investigate the relationship between verbal memory performance and white matter diffusivity in patients with PCA. We assessed white matter damage as a function of microstructural properties (fractional anisotropy, FA; mean diffusivity, MD). We predicted lower FA and higher MD within regions which are both consistently affected in PCA and strongly overlap with memory networks identified in healthy adults, namely the cingulum, ILF, SLF, splenium of the corpus callosum and uncinate fasciculus. More precisely, we hypothesised that white matter damage in tracts originating from these posterior structures would be specifically associated with poorer verbal memory retrieval.

## Materials and methods

### Participants

Eighteen participants fulfilling the diagnostic criteria for PCA (*n* = 18) were recruited from the Oxford Cognitive Disorders Clinic, Oxford, UK. Diagnoses were made by a senior behavioural neurologist (CB) and neuropsychologists (IB and SA) based on clinical, neuropsychological, and brain imaging features and in accordance with consensus criteria.^1^^,^^2^ These criteria included core features of simultanagnosia with or without optic ataxia or ocular apraxia, constructional apraxia, visual field defect, elements of Gestmann's syndrome or environmental disorientation, and the presence of the supportive features of alexia, ideomotor or dressing apraxia, or propopagnosia. Focal atrophy in the occipital and parietal lobes was confirmed in all patients with clinical MRI scans. Detailed grey matter assessments of the patients are reported in a previous voxel-based morphometry study.^12^ Neuropsychological assessment showed that patients presented with salient visuospatial and perceptual impairment, in line with the clinical phenotype (Table 3). Detailed memory assessments and MRI DTI data were available for 14 PCA patients. Scans from one PCA patient were excluded from the DTI analyses due to artefacts.

**Table 3:** Demographic and clinical characteristics of control and patient groups. Standard deviations are given in brackets.

	HC1	PCA	*P*-value
Demographics			
* N*	19	14	–
* *Age (years)	63.1 (6.3)	63.8 (7.0)	0.75
* *Education (years)	14.5 (2.2)	13.5 (2.3)	0.21
* *Gender (m: F)	8:11	8:6	0.39
* *Symptom duration	–	3.7 (1.9)	–
VOSP			
* *Dot count (max. 10)^a^	10.0 (0.0)	4.7 (3.4)	<0.001
* *Position discrimination (max. 20)^a^	19.6 (1.1)	13.5 (4.1)	<0.001
* *Cube analysis (max. 10)^a^	9.4 (1.3)	1.9 (2.4)	<0.001
RCFT			
* *Copy (max. 18)^a^	17.6 (0.7)	2.0 (3.0)	<0.001
* *Immediate Recall (max. 18)^a^	10.6 (3.7)	0.8 (1.0)	<0.001
* *Delayed recall (max. 18)^a^	10.5 (3.2)	0.2 (0.4)	<0.001
FCSRT-IR			
* *Immediate cued recall (max. 16)	14.8 (2.9)	14.1 (1.9)	0.13
* *Total recall (max. 48)	47.6 (1.0)	19.9 (8.7)	<0.001
* *Free recall (max. 48)	32.7 (4.6)	21.8 (5.1)	<0.001
* *Cued recall (max. 48)	14.9 (4.3)	41.7 (5.2)	<0.001
* *Cue sensitivity (%)	98.1 (4.8)	80.1 (14.2)	<0.001
Imaging cohort	**HC2**	**PCA**	***P*-value**
* N*	18	13	–
* *Age (years)	68.0 (6.2)	62.6 (13.7)	0.02
* *Education (years)	14.1 (3.5)	13.7 (1.9)	0.76
* *Gender (m: F)	11:7	7:6	0.69
* *Symptom duration (years)	–	3.7 (2.0)	–

FCSRT-IR, Free and Cued Selective Reminding Test with Immediate Recall; PCA, posterior cortical atrophy; RCFT, Rey Complex Figure Test; VOSP, Visual Object and Space Perception Battery.

a Missing data: Data of PCA patients was missing for some tests due to refusal or inability to complete. Reduced sample sizes were present for: RCFT Copy and Immediate Recall (*n* = 9), RCFT Delayed Recall (*n* = 8), VOSP Dot Count (*n* = 12), VOSP Position Discrimination (*n* = 10), and VOSP Cube Analysis (*n* = 9).

Nineteen healthy volunteers were recruited from the local community as a behavioural control group (herein referred to as healthy control group 1; HC1) for cross-sectional comparisons. Exclusion criteria were a history of psychiatric illness, head injury, cerebrovascular disease or current medication known to affect cognition. A separate group of 18 healthy control subjects (herein referred to as healthy control group 2; HC2) was recruited from the Oxford project to Investigate Memory and Ageing and the Memory and Amnesia Project, University of Oxford, UK (for further details of original cohorts see Refs.^12^^,^^14^^,^^64^^,^^65^). Control participants had no objective cognitive impairments, as indicated by scores within the normal range on the Addenbrooke's Cognitive Examination (ACE^66^^,^^67^) or the Mini-Mental State Examination (MMSE^68^). HC2 participants underwent DTI scanning on the same MRI scanner with identical sequences as the PCA group.^65^ Demographic and clinical characteristics of all participants are provided in Table 3. Within the imaging cohort, there was a significant difference in age between the PCA patient and HC2 groups. All neuroimaging analyses were therefore corrected for age. HC1 and HC2 groups did not differ in either age (*t*(32) = −2.02, *P *=* *0.052) or gender (*χ*^2^ (1, *N* = 35) = 0.24, *P *=* *0.621). Ethical approval was obtained from the National Research Ethics Service South Central—Hampshire B and Oxford C. All participants provided written informed consent in accordance with the Declaration of Helsinki.

### Memory assessment

Episodic memory was assessed using the Free and Cued Selective Reminding Test with Immediate Recall (FCSRT-IR^69^). In the study phase of this task, participants were presented with cards containing four black-and-white line drawings, as per standard administration of the task. Taking into account the visuo-perceptual impairments in PCA, the examiner additionally read the name of each item concurrent with the picture presentation and asked the participant to repeat it. This procedure ensured that visuo-perceptual difficulties did not obstruct encoding of the items. The examiner then provided a verbal semantic category cue (e.g. fruit) and the participant was asked to name the associated item (e.g. grapes). Once each of the items had been correctly identified, immediate cued recall of the four items was assessed by verbally presenting the four category cues. If an item was not recalled the card was shown again and the categories verbally repeated. The participant was asked to name and select the relevant item, after which immediate cued recall was reattempted. When the participant had recalled all four items, the next set of items was presented as above, until all 16 items had been learned in this way. Following the study phase, there were three recall trials, each involving two minutes of spontaneous free recall followed by cuing with the semantic category for words that were not retrieved. Scores were recorded for Immediate Cued Recall (number of items recalled directly after presentation), Free Recall (number of items spontaneously recalled), Cued Recall (number of items retrieved with cues), Total Recall (number of items retrieved with or without cues), and Cue Sensitivity (efficacy of semantic category cues in facilitating recall) calculated as (Total Recall—Free Recall)/(48—Free Recall). Statistical analyses were undertaken with the following metrics: Immediate Cued Recall, to assess encoding processes; Total Recall scores, to investigate delayed memory; and Free Recall and Cue Sensitivity to assess memory performance requiring high and low levels of attentional control, respectively.

### DTI data acquisition and analyses

MRI data were acquired with a 3T Siemens Trio scanner using a 32-channel head coil. Sixty non-collinear diffusion directions (*b *=* *1000 s/mm^2^) and five non-diffusion (*b* = 0 s/mm^2^) volumes were obtained using a transverse 2D echo planar DTI sequence (repetition time = 9300 ms; echo time = 94 ms; slice thickness = 2.5 mm; voxel size = 2.0 mm × 2.0 mm × 2.0 mm; FOV = 192 mm). Sixty-five axial slices covering whole brain were acquired for each participant.

All imaging data were analysed using the FMRIB Software Library (FSL^70^; Version 5.0.10). Pre-processing steps of diffusion-weighted images included correction for participant movements and eddy currents with the ‘eddy’ command.^71^ Maps of FA and MD were derived by fitting the diffusion tensor model within each voxel using FMRIB’s Diffusion Toolbox (FDT). Voxel-wise statistical analyses of the FA and MD data were carried out using tract-based spatial statistics (TBSS^72^). All participants’ FA data were aligned into a common space using the nonlinear registration tool FNIRT,^73^ which uses a b-spline representation of the registration warp field.^74^ Next, the mean FA image was created and thinned to create a mean FA skeleton representing the centres of all tracts common to the participant group. Each participant's aligned FA data were then projected onto the skeleton. The final non-linear warp and skeleton projection procedures were then repeated with the MD data.

The PCA and HC2 groups were compared with a two-sample unpaired *t*-test using the ‘randomise’ tool run on a whole-brain mask.^75^ Next, within the resulting regions of significant difference between the two groups, correlational analyses were run to test for specific associations between FA and MD measures and the degree of memory impairment in PCA patients (no FCSRT-IR data were available from the HC2 control group). For this purpose, binary masks were created from the group comparison at a threshold of *P *<* *0.1 and the ‘randomise’ tool used to test for correlations between FCSRT-IR scores and FA and MD measures within the binary masks, adding age as a covariate of no interest to the model. For all analyses, a threshold-free cluster enhancement (TFCE) *P *<* *0.05 correction was applied, with the number of permutations set at 5000. To ascertain the specificity of correlations with the memory domain, analyses were re-run with inclusion of patients' visual function as a co-variate, as estimated from scores on the visual subdomain of Addenbrooke's Cognitive Examination.

Finally, given the relatively modest size of the PCA sample, we applied region-of-interest (ROI) analyses to complement and confirm the results from the whole-brain TBSS approach. Seven ROIs which have consistently been identified as showing degeneration with PCA in prior studies (see Table 1) *and* are associated with memory performance (see Table 2) were defined for further investigation within the patient cohort. These were the right and left SLF, right and left ILF, right and left cingulum, and splenium of the corpus callosum. To create the ROIs, masks of the regions were extracted based on the JHU ICBM-DTI-81 white matter labels and JHU white-matter tractography atlas within the FSL atlas tool and multiplied with the mean skeleton. The average FA and MD values were extracted for each participant and region, and correlations with memory scores were assessed using statistical analyses as described below.

### Post-hoc tractography analyses

After identification of the regions associated with memory performance in PCA patients, we carried out post-hoc tractography analyses to determine the associated white matter pathways in healthy controls.

Fibre orientation distributions were computed at each voxel for two fiber directions with the bedpostx tool.^76^^,^^77^ Probtrackx2 was used to perform probabilistic tractography, with the number of individual streamlines set at 5000 and a curvature threshold of 0.2.^76^^,^^77^ We generated a binary mask from voxels correlated with memory scores within the patient group in TBSS analyses (*P *<* *0.05). This mask was projected onto each control participant's native DTI space and used as a seed for tractography, with resulting distributions being transformed back into MNI space. For each participant, results were divided by the total number of streamlines and thresholded at 0.01 to exclude low-probability pathways. Finally, thresholded distributions were binarized and averaged across individuals to obtain a group map.

### Statistical analysis

All statistical analyses were performed using R software (version 3.6.0). Between-group differences on continuous demographic variables and task performance were assessed with two-tailed *t*-tests or Mann–Whitney *U*-tests for non-normal distributions, and comparisons of categorical variables were performed using *χ*^2^ tests.

The relationship between episodic memory scores and FA or MD values extracted from regions of interest was assessed using Spearman's partial correlation analyses adjusted for age. Bonferroni corrections were performed to control for Type I errors.

### Data availability

The data are not publicly available due to privacy and ethical restrictions.

## Results

### Memory assessment

There was no significant difference in Immediate Cued Recall scores between PCA patients and HC1 (*U *=* *184, *P *=* *0.131), suggesting that encoding of verbal items was spared in PCA. However, PCA patients obtained significantly lower scores on Total FCSRT-IR (*U *=* *266, *P *<* *0.001), Free Recall (*t*(31) = 6.32, *P *<* *0.001), and cue sensitivity (*U *=* *234, *P *<* *0.001) compared to the HC1 group. Semantic categories continued to support word retrieval in PCA, as indicated by a cue sensitivity of 80.1%.

### Whole-brain voxel-based comparison between PCA and healthy controls

**Figure 1:**
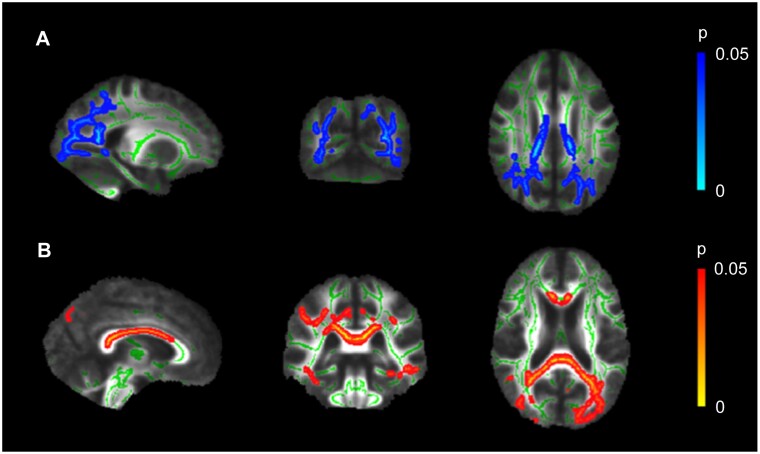
**Fractional anisotropy and mean diffusivity differences between groups** (**A**) Significant reduction of FA (blue) in PCA patients compared to controls (*x* = −29, *y* = 59, *z* = 101). (**B**) Significant increase of MD (red) in PCA patients compared to control participants (*x* = −3, *y* = −35, *z* = 18). MNI coordinates are in millimetres and the skeletonised results have been thickened to improve visibility of the affected white matter regions for illustrative purposes.

The group comparison demonstrated lower FA and higher MD in the forceps major, ILF, inferior fronto-occipital fasciculus, SLF, anterior thalamic radiation, cingulum, and body and splenium of the corpus callosum in PCA patients relative to HC2 participants (Fig. 1). There were no increases in FA or decreases in MD in PCA patients relative to the HC2 group.

### Voxel-based correlations of FCSRT-IR with measures of white matter integrity in PCA

**Figure 2:**
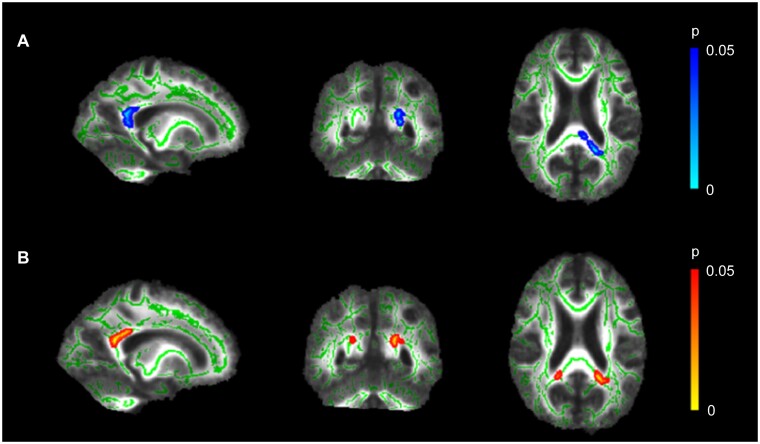
**White matter microstructure in the splenium associated with memory retrieval.** Higher FCSRT-IR scores on the Free Recall component were associated with (**A**) higher FA in left-sided splenium (*x* = −19, *y* = −50, *z* = 19) and (**B**) lower MD in bilateral splenium of the corpus callosum (*x* = −18, *y* = −49, *z* = 21). MNI coordinates are in millimetres and the skeletonised results have been thickened to improve visibility of the affected white matter regions for illustrative purposes.

Within the PCA patient group, a significant positive correlation was observed between FA values in the splenium and Free Recall scores on the FCSRT-IR in TBSS analyses (Fig. 2A). In addition, a negative correlation for splenium MD and Free Recall was found (Fig 2B). Including visual functioning as a covariate of interest decreased significance but did not change the overall pattern of results. No further significant associations were observed for either FA or MD measures and Immediate Cued Recall, Cue Sensitivity or Total Recall scores.

### ROI analyses of FCSRT-IR performance

Consistent with the whole-brain analyses, the ROI analyses indicated that there was a negative relationship between FCSRT-IR Free Recall scores and MD values in the splenium of the corpus callosum (see Table 4). No significant correlations were observed for MD or FA in any of the ROIs with FCSRT-IR Immediate Cued Recall, Cue Sensitivity or Total Recall scores.

**Table 4:** Age-adjusted Spearman’s partial correlations between FCSRT-IR and ROI white matter microstructure in PCA patients.

	Immediate cued recall	Free recall	Total recall	Cue sensitivity
FA				
* *Left SLF	−0.05	−0.05	0.06	0.18
* *Right SLF	−0.39	−0.16	−0.34	−0.30
* *Left ILF	−0.35	−0.33	−0.23	−0.12
* *Right ILF	−0.39	−0.43	−0.42	−0.39
* *Left cingulum	0.06	0.36	0.20	0.19
* *Right cingulum	0.37	0.44	0.43	0.34
* *Splenium	0.16	0.48	0.30	0.35
MD				
* *Left SLF	−0.11	0.44	0.30	0.08
* *Right SLF	0.00	0.28	0.26	0.31
* *Left ILF	0.36	−0.31	−0.29	−0.35
* *Right ILF	0.25	−0.06	−0.07	−0.17
* *Left cingulum	0.06	−0.22	0.03	0.08
* *Right cingulum	0.20	−0.04	0.09	0.20
* *Splenium	−0.40	−0.71^a^	−0.57	−0.46

FA, fractional anisotropy; MD, mean diffusivity; ILF, inferior longitudinal fasciculus; SLF; superior longitudinal fasciculus. *r* represents Spearman's correlation coefficient.

a *P *=* *0.038, corrected for multiple comparisons.

### Post-hoc tractography

**Figure 3:**
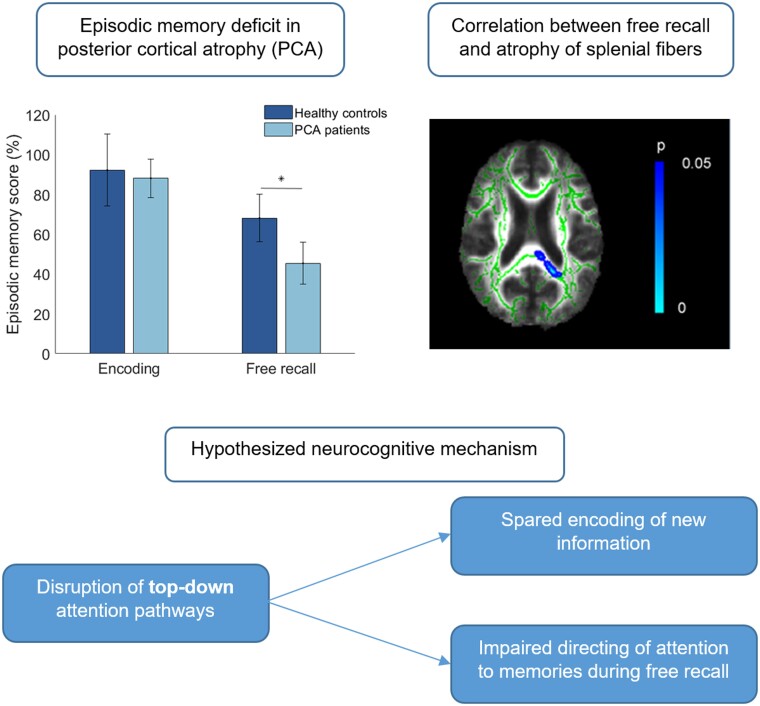
**Average probabilistic tractography results in healthy adults seeded from the splenial mask derived from PCA patients.** Thresholded streamlines (red) passed through the thalamic radiations (light blue) and retrolenticular internal capsule (dark blue) as defined by the JHU-DTI atlas.

Splenial voxels significantly associated with FCSRT-IR Free Recall scores in PCA were used as a seed mask for probabilistic tractography in the healthy control group. As shown in Fig. 3, streamlines from the voxels of interest in the control participants passed bilaterally through the thalamic radiations and the retrolenticular part of the internal capsule.

## Discussion

In this study, PCA patients presented with impaired free recall and relatively spared cued retrieval on a task of verbal episodic memory. In line with previous studies,^12^^,^^64^ these results are suggestive of a different cognitive basis for the memory dysfunction associated with PCA compared with the typical storage deficits observed in amnestic Alzheimer’s disease. DTI showed that poor memory retrieval was specifically associated with reduced FA and increased MD of posterior regions of the cortex, namely the splenium of the corpus callosum.

The splenium is highly interconnected with posterior parietal, occipital and temporal regions of the brain, all adversely affected in PCA.^78^^,^^82^ As part of the corpus callosum, the splenium forms a major commissural area of the brain, with the dorsal region of the splenium forming connections between bilateral parietal lobes and the ventral regions connecting the temporal cortices.^80^^,^^81^ Degeneration of the splenium can therefore result in disconnection of these posterior regions of the cerebral cortex, with resulting disruption of cognitive functioning.^83^ The splenium of the corpus callosum has consistently been found to be vulnerable in PCA.^3^^,^^44^^,^^45^^,^^50^^,^^52^^,^^53^ Neuropsychological studies have linked symptoms of visuospatial neglect to splenial lesions^84^ and marked perceptual difficulties in patients with tumours of the splenium,^85^ and it is therefore plausible that the splenial changes contribute to the characteristic visuospatial deficits which define PCA. The splenium has not previously been considered in connection to memory impairments in PCA. The memory literature, however, presents a strong evidence base for its role in episodic memory. In healthy older adults, integrity of the splenial fibres has been shown to relate to measures of immediate and delayed verbal memory recall.^60^^,^^61^ In Alzheimer’s disease, decreases in FA of the posterior corpus callosum were associated with poor delayed verbal recall and visual memory.^86^ Of particular relevance, a study of patients with tumours of the splenium observed memory impairments alongside visuospatial deficits,^85^ presenting a symptom profile similar to PCA.

From a mechanistic point of view, we propose that there are at least three different pathways through which splenial degeneration could influence memory performance. A first explanation is that since the corpus callosum plays an integral role in transferring information between the two hemispheres, damage to this region results in slowed information processing. Corpus callosum integrity has been found to significantly predict processing speed across various neuropsychological tasks.^87^^,^^88^ One study even proposed that reduced processing speed mediates the relationship between age and episodic memory decline.^89^ Slowed processing speed could therefore be hypothesized to be the driving factor in the association of the splenium with memory processes. However, this theory cannot explain the presentation of impaired memory retrieval with relatively preserved encoding skills observed in PCA. Reduced processing speed would be expected to interfere more generally with cognitive processes^90^ rather than being limited to retrieval mechanisms.

A second possibility is that the effects of splenium integrity are indirect and mediated by regions which are more strongly implicated in memory retrieval. For instance, Rudge and Warrington^85^ suggested that memory impairments in patients with tumours of the splenium are due to consequent damage to the fornix. The fornix forms a major efferent pathway from the medial temporal lobe, with posterior fibres projecting to the cerebral cortex and brainstem through the splenium of the corpus callosum.^91^ As such, it provides an important connection between the hippocampus and posterior regions affected in PCA. It is well-established that the fornix is linked to memory performance.^54^^,^^57^^,^^58^^,^^92–95^ Metzler-Baddeley et al.^62^ showed that FA values in the fornix were positively correlated with Free Recall and Total Recall measures of the FCSRT-IR in healthy older adults. In the present study, there was no evidence for damage to the fornix in patients compared with healthy controls, indicating that white matter integrity of this structure is not directly linked to memory performance in PCA. However, one might propose that damage of the splenium results in reduced functioning of connected structures such as the fornix, which in turn affects memory. Additional research is warranted to further explore potential interactions between the splenium and the fornix.

Finally, memory impairments may stem from disruption of attentional processes. The corpus callosum has been found to play a modulatory role in visuospatial attention networks. In particular, microstructural properties of the splenium have been related to the orienting component of attention in healthy adults.^96^ Reductions of white matter connectivity in posterior corpus callosum are correlated with reduced quality of orienting of attention.^97^ Furthermore, lesions of and reduced FA in the splenium have been associated with hemispatial neglect,^84^ a disorder which at its core is thought to involve impaired orienting of attention. Congruent with the AtoM model,^24^ it is possible that such changes in visual attention can be extrapolated to memory performance, that is, damage of the splenium may lead to difficulties in focussing on relevant stimuli, whether this concerns external (i.e. visual) or internal (i.e. memories) information. In the context of previous investigations of memory in PCA, this explanation is particularly compelling. Voxel-based morphometry and resting-state fMRI analyses provide converging evidence for a critical role of parietal attention networks as opposed to classic memory circuitry in retrieval dysfunction.^12^^,^^14^ Of particular relevance, voxel-based morphometry analyses of the same patient cohort found largely spared temporal regions, with significant correlations between autobiographical memory performance and atrophy of lateral parietal cortex but not hippocampal damage.^12^ Notably, post-hoc tractography analyses in the present study demonstrated that the splenial region associated with memory performance in PCA was connected to the internal capsule in healthy adults, a structure which has previously been linked to orienting of attention.^96^^,^^97^ These results are suggestive of a specific disruption of white matter pathways supporting attentional processing, rather than being caused by reduced integrity of connections with regions directly implicated in memory function (e.g. fornix). Crucially, the distinction between bottom-up and top-down attentional processes central to the AtoM model could also account for the pattern of impaired free recall in the face of comparatively intact cue sensitivity. Within this framework, specific disruptions of top-down attention would result in difficulties with spontaneously directing attention to memories, whereas patients could fare better when provided with external input (i.e. category cues). An attentional mechanism driving memory impairments in PCA could therefore provide a unifying account for the findings across these MRI modalities. Imaging studies explicitly testing for non-spatial attentional control processes in relation to the corpus callosum are needed to clarify this potential role for splenial fibres within the AtoM account of episodic memory.

The findings presented here have several key clinical implications. From a diagnostic perspective, the finding of splenial contributions to memory deficits that may be linked to attentional processes can help to inform which memory assessments are used to profile PCA patients. Although memory is not systematically examined in PCA, a body of research has begun to delineate the nature of memory disruption this group, highlighting it as a common supporting feature of the early clinical phenotype.^2^ Targeted memory assessment can therefore be used as a supplementary marker to increase diagnostic accuracy. Our results suggest that, although PCA patients are able to store verbal information, they have difficulties in recalling the items without external prompts. Direct comparisons between PCA and typical Alzheimer’s disease patients show that the former tend to learn word lists more rapidly, but that the two groups are equally impaired on delayed recall measures.^12^ Tasks which clearly distinguish between encoding and retrieval difficulties may therefore be particularly useful in differentiating PCA from other dementia phenotypes. Potential examples include the FCSRT-IR used here, as well as classic list learning tasks such as the Rey Auditory Verbal Learning Test (RAVLT^98^) and California Verbal Learning Test (CVLT^99^). Our findings also have implications for the identification and development of effective therapeutic approaches to support memory functioning in PCA. For example, if the role of the splenium in attentional components of memory is further supported, PCA patients may derive greater benefit from strategies aiming to help direct attention and minimise distractions, rather than classic compensatory aids to memory. The delivery of appropriate memory support is particularly important as deficits have been associated with greater difficulties in performing activities of daily living^100^^,^^101^ and increased caregiver burden^102^ in dementia, and specifically in PCA.^103^

Several limitations to the present study must be acknowledged. First, the neuropsychological battery did not incorporate detailed assessments of different aspects of memory or attention. Whilst we cautiously suggest that attentional processes may lie at the root of the memory deficits in PCA, additional testing with both attention and memory tasks will be essential to directly test this proposal as well as competing hypotheses. Despite this shortfall, the FCSRT-IR has a well-evidenced base and is widely posited as a go-to memory assessment in dementia.^104^ We are confident, therefore, that our findings remain a robust addition to our understanding of memory in PCA. Second, although the sample size of the patient group was not small given the relative rarity of the disorder, a larger sample would provide more power to detect subtle associations between white matter and memory performance. Future studies using a larger sample size would therefore be valuable in confirming and extending the present findings. Third, our hypotheses regarding the association between white matter integrity and memory in PCA focussed only on those mechanisms which are reliably linked to both the splenium and memory, and are therefore non-exhaustive. For example, an intriguing new line of work on autobiographical memory in PCA has uncovered a relationship between visual imagery and memory deficits in posterior parietal regions.^12^^,^^105^ It would be of interest to explore whether such associations extend to episodic memory tasks such as the FCSRT-IR, which in contrast to autobiographical memory assessments do not hinge on the retrieval of rich contextual information. Finally, as the healthy control cohort undergoing MRI scanning (HC2) did not complete the FCSRT-IR, it was not possible to conduct between-group comparisons for the relationship between DTI measures and memory performance. It is therefore unclear whether the proposed contributions of the splenium to memory extend to the wider population. As the HC1 control group performed near ceiling on the FCSRT-IR, it is unlikely that healthy adults show sufficient variability on the task to detect significant associations with white matter integrity. However, further explorations with other patient groups (e.g. PCA versus typical Alzheimer’s disease) are warranted to directly contrast the pathways associated with memory deficits across types of dementia.

## Conclusions

In summary, the results of this study show that memory deficits in PCA are specifically associated with diffusivity changes in the splenium of the corpus callosum. Potential mechanisms by which the splenium could contribute to retrieval processes include indirect effects via the limbic system or attention networks, with the latter presenting a particularly compelling line of future inquiry. The findings have significant translational value in increasing our understanding of the broader clinical phenotype of PCA, aiding in diagnostic assessment and signposting to tailored post-diagnostic support.

## Abbreviations

DTI =: diffusion tensor imaging
FA =: fractional anisotropy
FCSRT-IR =: Free and Cued Selective Reminding Test with Immediate Recall
ILF =: inferior longitudinal fasciculus
MD =: mean diffusivity
PCA =: posterior cortical atrophy
ROI =: region-of-interest
SLF =: superior longitudinal fasciculus
TBSS =: tract-based spatial statistics
