# Corrigendum to: Splenial white matter integrity is associated with memory impairments in posterior cortical atrophy

**DOI:** 10.1093/braincomms/fcab129

**Published:** 2021-06-30

Margot Juliëtte Overman, Giovanna Zamboni, Christopher Butler and Samrah Ahmed; Splenial white matter integrity is associated with memory impairments in posterior cortical atrophy. *Brain Communications* 2021; 3(2): doi:10.1093/braincomms/fcab060.

In the originally published version of this manuscript, the graphical abstract was mistakenly used as Figure 3.

The correct Figure 3 is:

**Figure 3:**
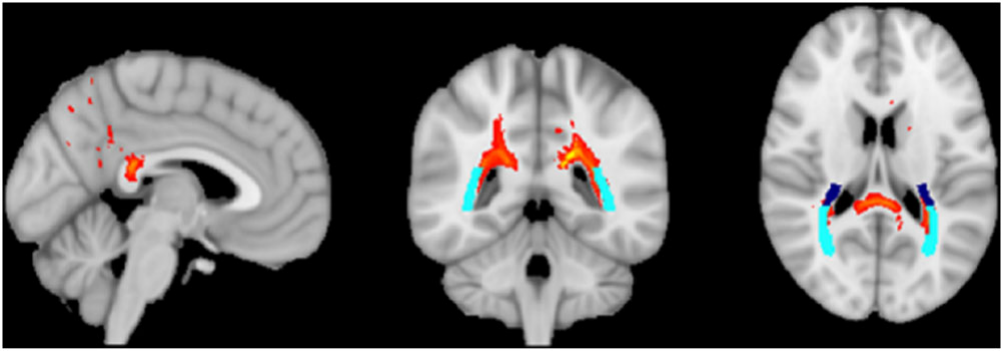
**Average probabilistic tractography results in healthy adults seeded from the splenial mask derived from PCA patients**. Thresholded streamlines (red) passed through the thalamic radiations (light blue) and retrolenticular internal capsule (dark blue) as defined by the JHU-DTI atlas.

This error has been corrected online.

